# Treating Venous Thromboembolism Post Intracranial Hemorrhage: A Case Report

**DOI:** 10.7759/cureus.6746

**Published:** 2020-01-22

**Authors:** Aman N Ajmeri, Kamran Zaheer, Colin McCorkle, Ahmed Amro, Bisher Mustafa

**Affiliations:** 1 Internal Medicine, Marshall University, Joan C. Edwards School of Medicine, Huntington, USA; 2 Cardiology, Marshall University, Joan C. Edwards School of Medicine, Huntington, USA

**Keywords:** factor v leiden, ich, anticoagulation, ivc filter, pe, cerebral cavernous malformation, ccm-related bleeding, cavernous angioma, intracranial hemorrhage, pulmonary embolism

## Abstract

Venous thromboembolism (VTE) is a significant issue occurring due to genetic, acquired and circumstantial risk factors. Treatment is according to the clinical situation and judgment for long term anticoagulation based on individual risk. Anticoagulation after a history of a hemorrhagic stroke poses a therapeutic dilemma. We present a case of a 68-year-old male who presented with right-sided chest pain and shortness of breath. Workup included a CT that was positive for multiple right-sided pulmonary emboli (PE). The patient has a past medical history of Factor V Leiden Mutation, recurrent PE, and deep vein thrombosis (DVT). Two months prior he was diagnosed with a 1.3-cm intracranial hemorrhage (ICH) from multiple cavernous angiomas. At that time his warfarin was discontinued and an inferior vena cave (IVC) filter was placed. Facing the recent ICH and now multiple and recurrent PE, it was decided to resume anticoagulation based on ICH location. ICH from a deep source is likely a better characteristic that favors a resumption of anticoagulation. Our case will highlight that IVC filters cannot be solely relied upon in patients that are at high risk for thrombotic events with underlying genetic thrombophilia.

## Introduction

Venous thromboembolism (VTE) is a pathology that starts as a deep vein thrombus with subsequent dislodging and movement of thrombus to another location. Thrombus movement to the lungs is most common and referred to as pulmonary embolus. It is a significant problem that affects approximately one in 1000 persons per year. VTE usually occurs as a result of circumstantial risk factors and/or genetic risk factors. Approximately half of the patients presenting with VTE have a concurrent underlying thrombophilia disorder [1]. Factor V Leiden is the most widely recognized genetic risk factor predisposing for VTE and it is present in approximately 20-25% of patients with VTE [2,3]. Unfortunately, it may go undiagnosed as there are no clinical features specific for factor V Leiden thrombophilia; it is usually only diagnosed post VTE. Patients with Factor V Leiden deficiency are treated according to the clinical situation and may receive long-term anticoagulation therapy based on individual risk and clinical judgment [1].

Treatment of VTE requires three months of anticoagulation for proximal deep vein thrombosis (DVT), symptomatic distal DVT and pulmonary emboli (PE). Surveillance with serial ultrasound over a two-week period may be considered in patients with a high risk of bleeding, negative D-Dimer level, asymptomatic presentation, or without risk factors for extension. Duration of therapy and the possibility of indefinite anticoagulation should be individualized based upon risk factors such as unprovoked proximal DVT, recurrent DVT or multiple risk factors [4]. VTE associated with Factor V Leiden mutation is treated similarly to the general population with the same treatment duration considerations. Indefinite anticoagulation is highly recommended for DVTs that are unprovoked, life threatening, in an abnormal location and for recurrent episodes of VTE. Testing for Factor V Leiden is indicated for young patients with VTE, if a VTE occurs in an unusual location and for a member of a thrombophilic family. Testing is not indicted for a first provoked episode of VTE and in patients greater than 50 years old [5]. Anticoagulation poses a challenge when a patient suffers from an intracranial hemorrhage (ICH). Determining whether anticoagulation should be given post ICH presents a tricky conundrum when weighing the risk of a thrombotic event like PE or stroke versus risking another ICH. We present a case of a patient with a factor V Leiden mutation with a past medical history of recurrent DVTs and PEs, and an intracerebral hemorrhage from a cavernous angioma. The patient developed numerous pulmonary emboli after being taken off anticoagulation.

## Case presentation

**Figure 1:**
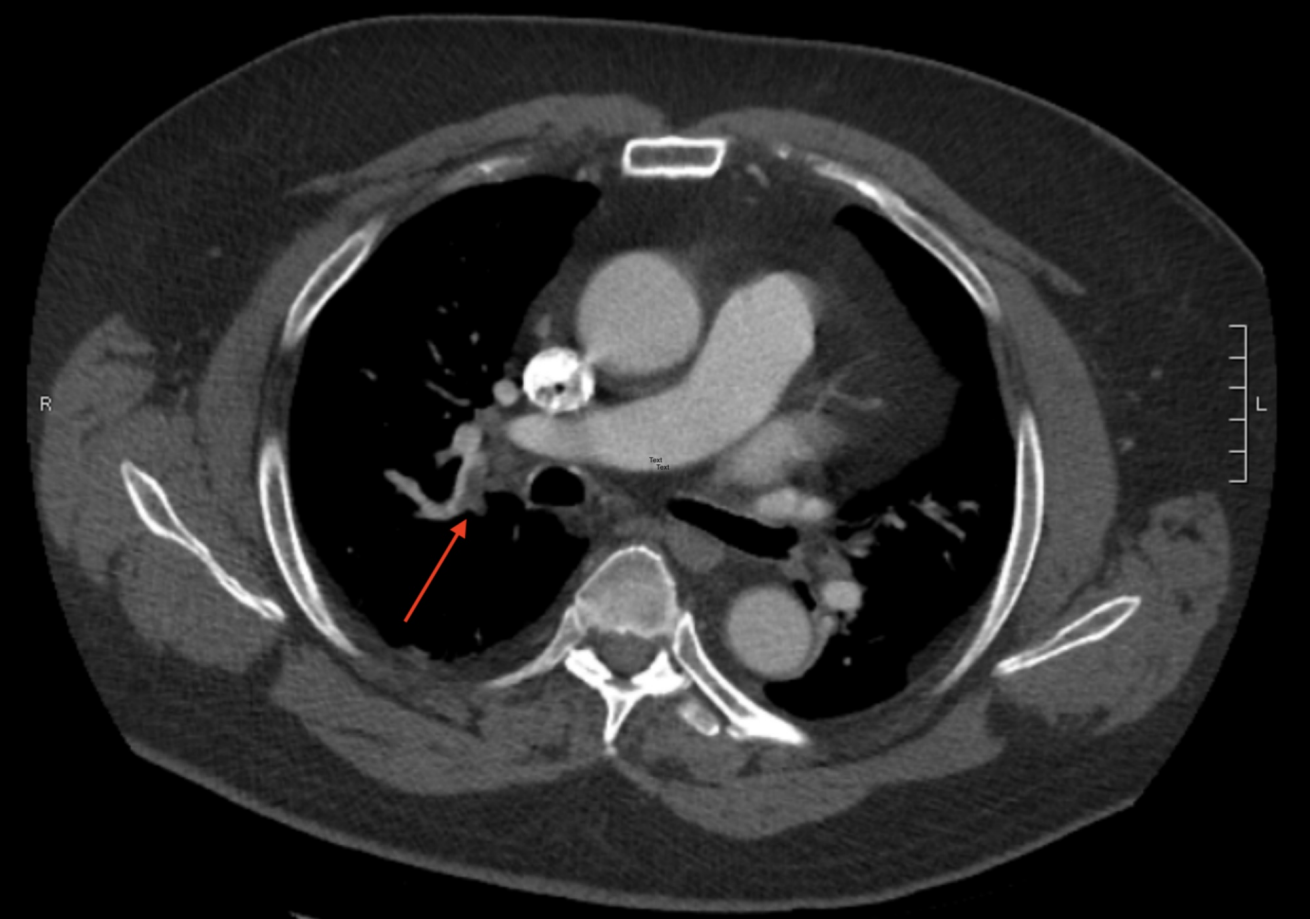
PE protocol CT scan showing a PE in the right pulmonary artery. PE: Pulmonary Embolism

**Figure 2:**
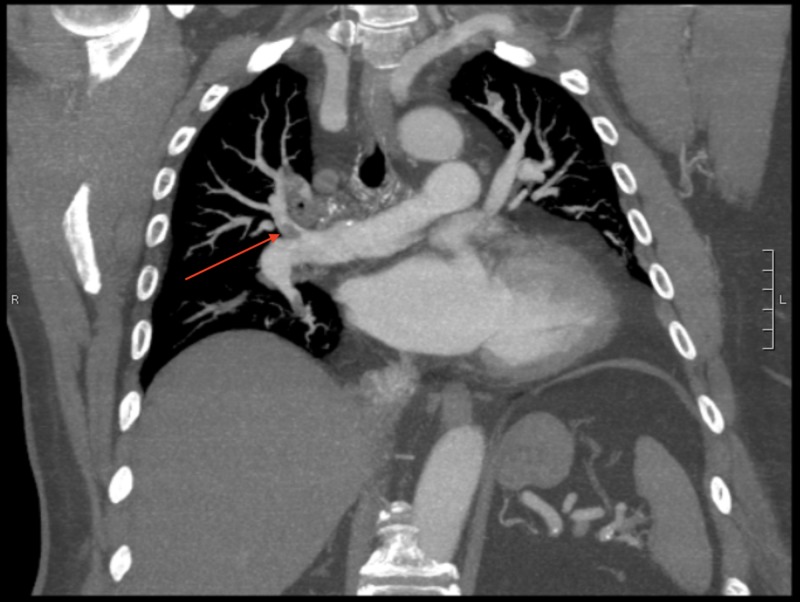
PE protocol showing right-sided PE in the right pulmonary artery. PE: Pulmonary Embolism

A 68-year-old male presented to the emergency department with ongoing right-sided chest pain and shortness of breath for the past four hours. He described the chest pain as a non-exertional, sharp pain with an intensity of 7/10 that was not relieved or aggravated by other factors. On arrival the patient was tachypneic with a respiratory rate of 20; all other vitals were stable. Electrocardiogram (EKG) was unremarkable and troponins were negative. Suspecting a VTE, CT Pulmonary Embolism protocol was performed, and the result was positive for right-sided PE with multiple associated emboli in the right lower lobe and right upper lobe pulmonary arteries (Figures 1-2). D-dimer was elevated at 4.67 ug/ml, and labs were all within normal limits including pro-BNP and troponin.

**Figure 3:**
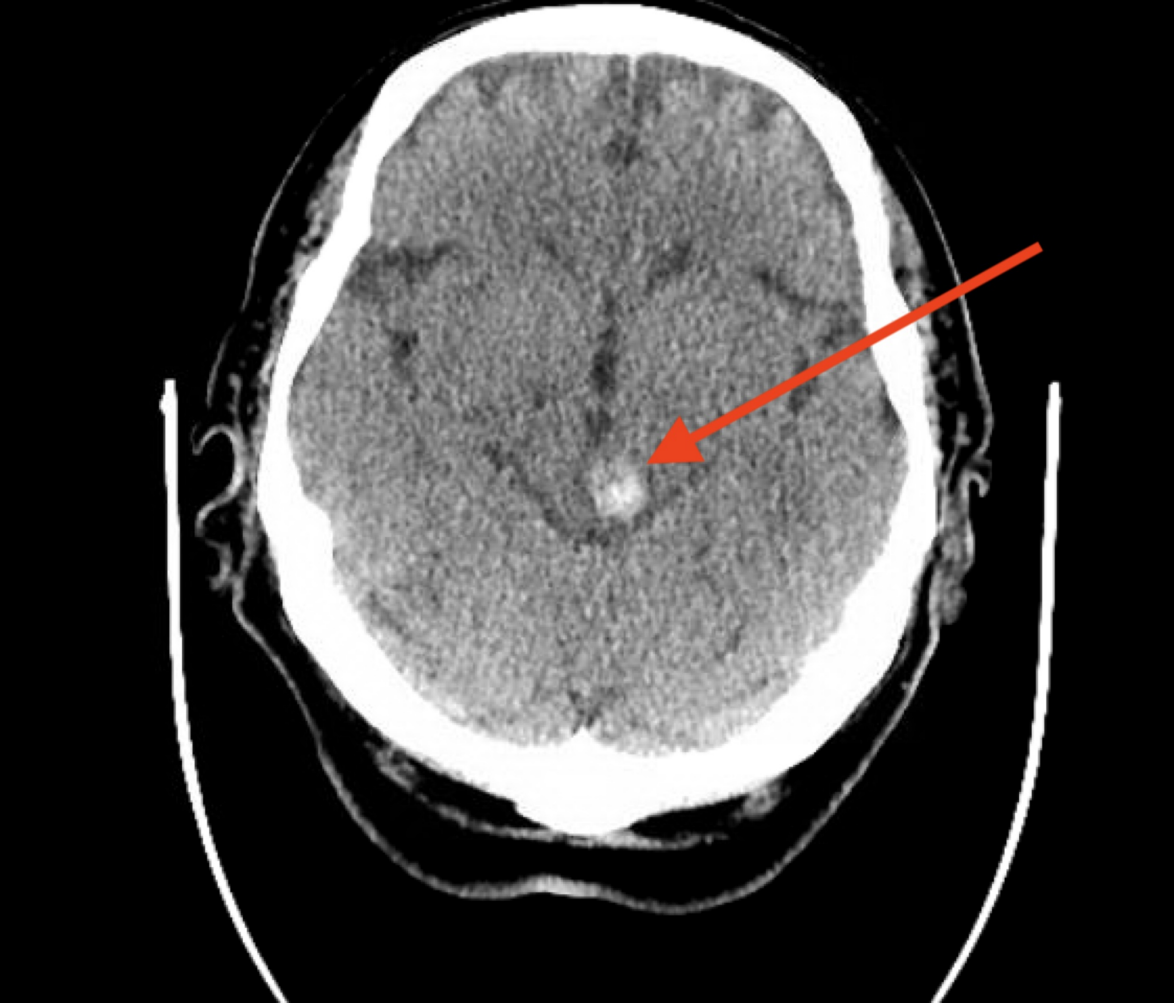
This is a CT without contrast at the time of presentation for neurologic deficits showing a rounded focus of varying hyperdensities suggesting hemorrhage of varying durations including acute and subacute hemorrhagic components.

**Figure 4:**
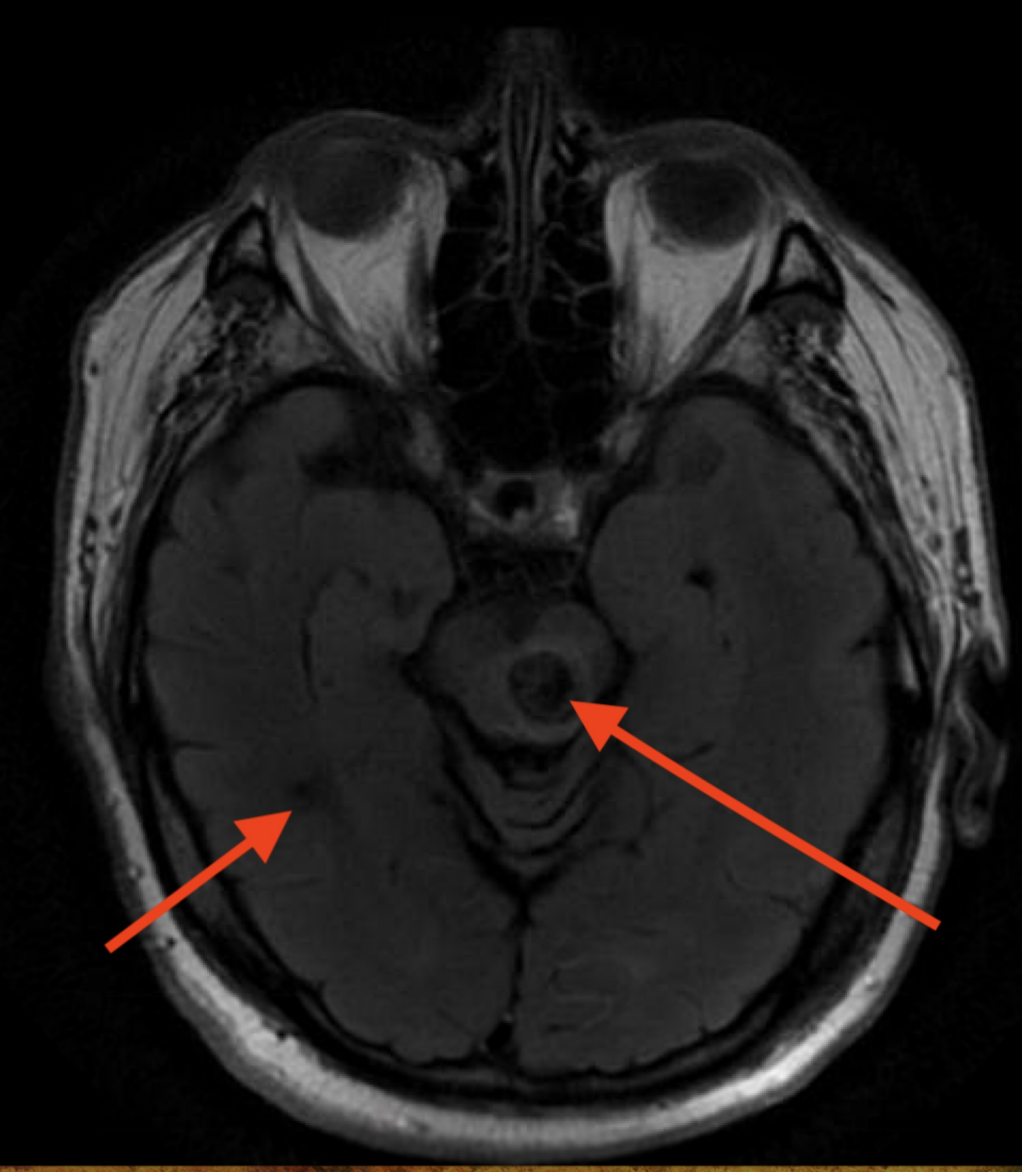
MRI FLAIR showing large hemosiderin depositions in the areas of cavernomas. FLAIR: Fluid-attenuated inversion recovery

**Figure 5:**
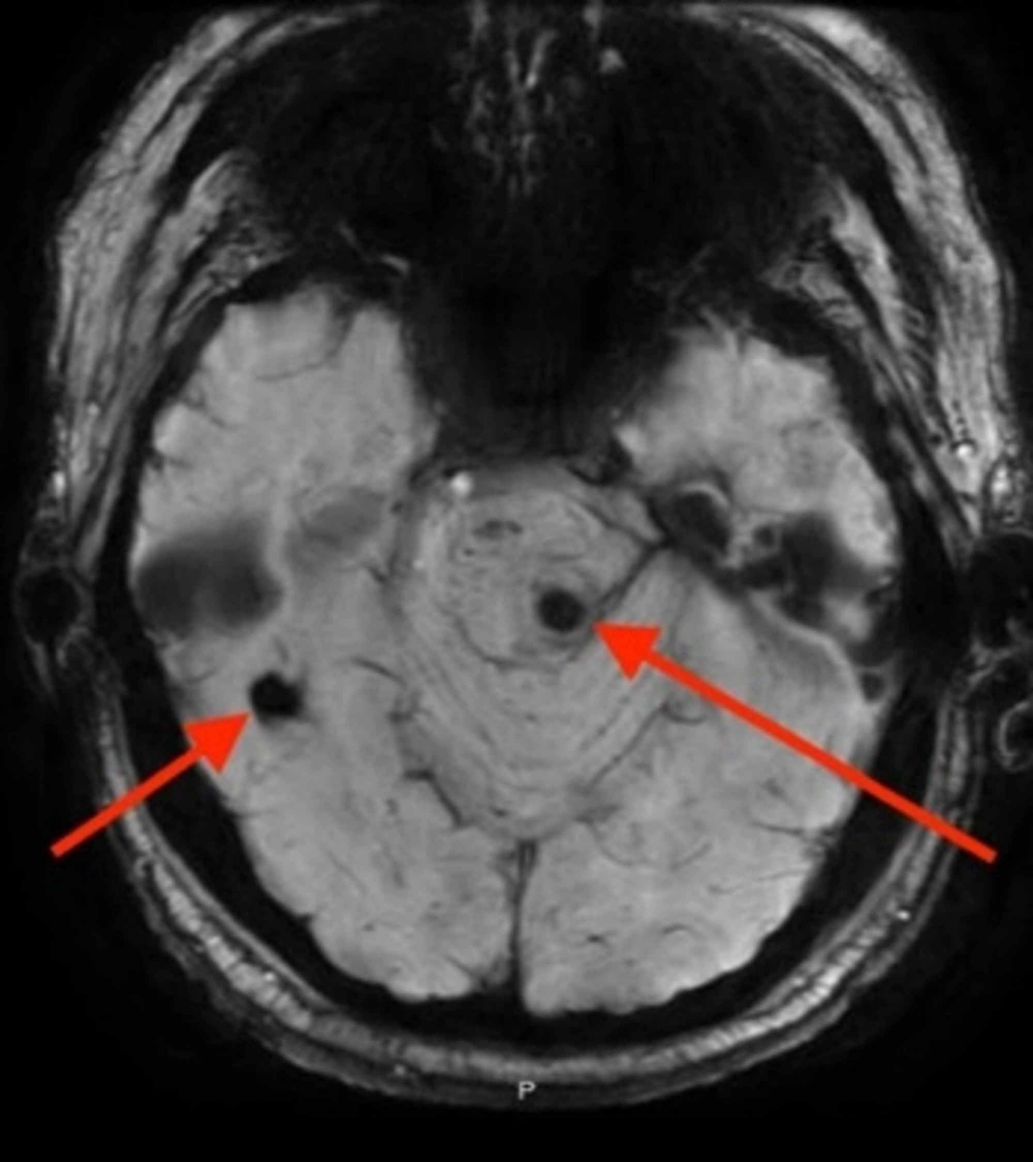
MRI SWI sequence showing multiple areas of hemosiderin deposition consistent with multiple CCMs. SWI: Susceptibility-Weighted Imaging; CCMs: Cerebral Cavernous Malformations.

**Figure 6:**
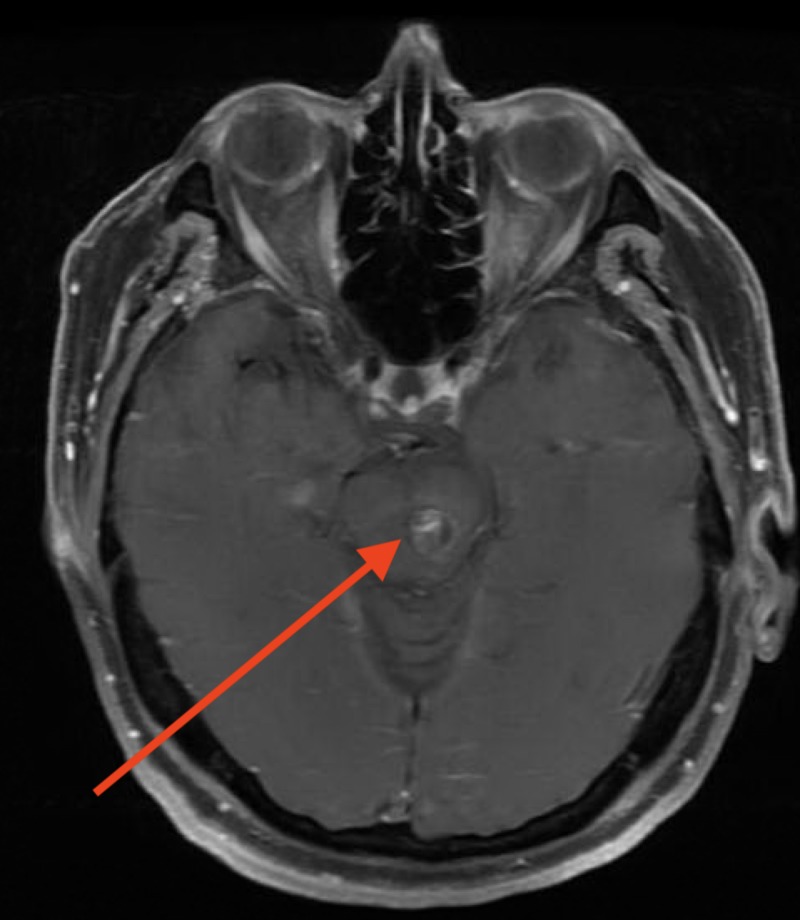
T1 post contrast image showing patchy ring enhancement associated with the hemorrhagic lesion consistent with a cavernous malformation.

**Figure 7:**
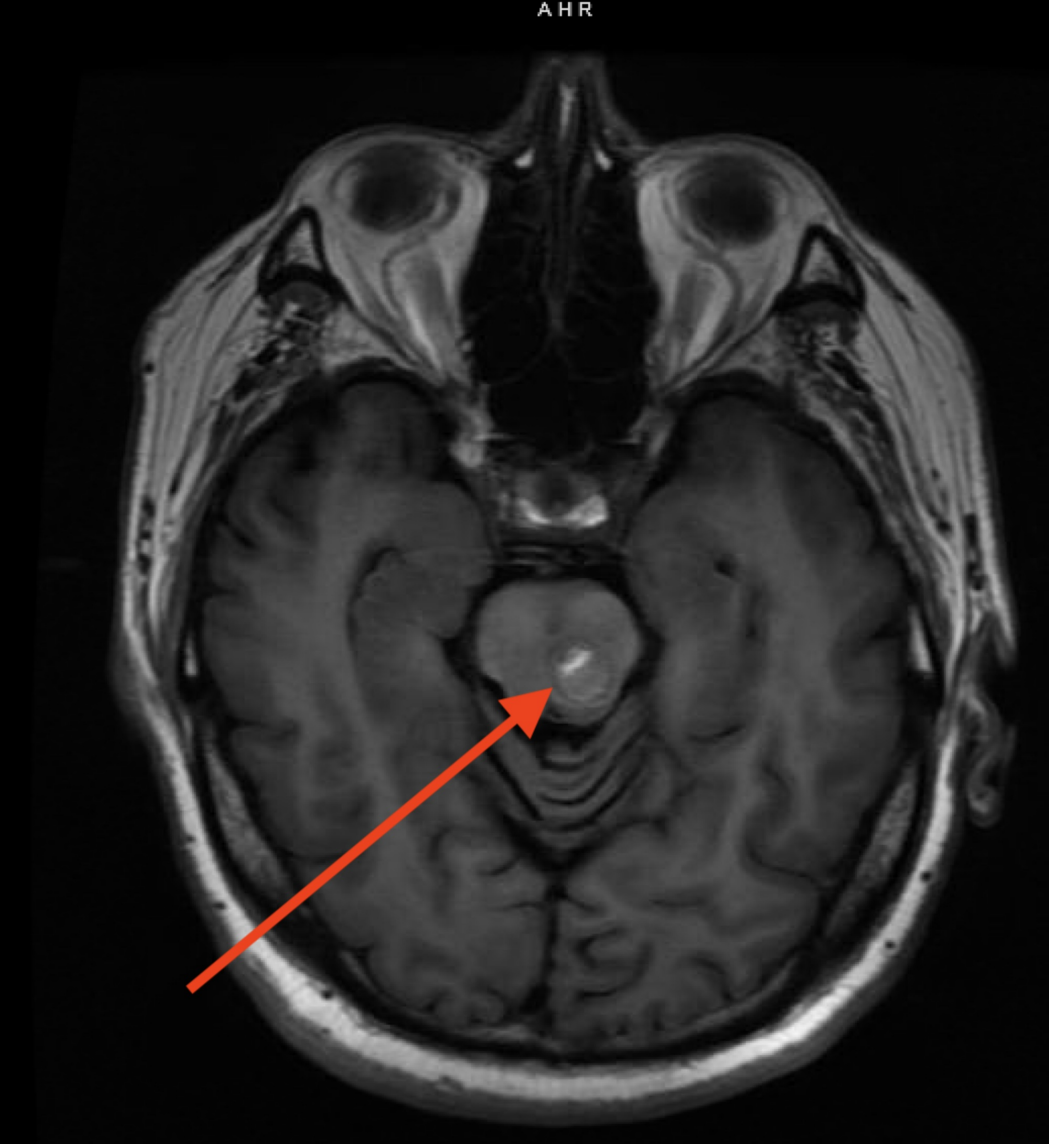
T1 MRI with arrow pointing towards hyperintense signal within angioma cavity.

**Figure 8:**
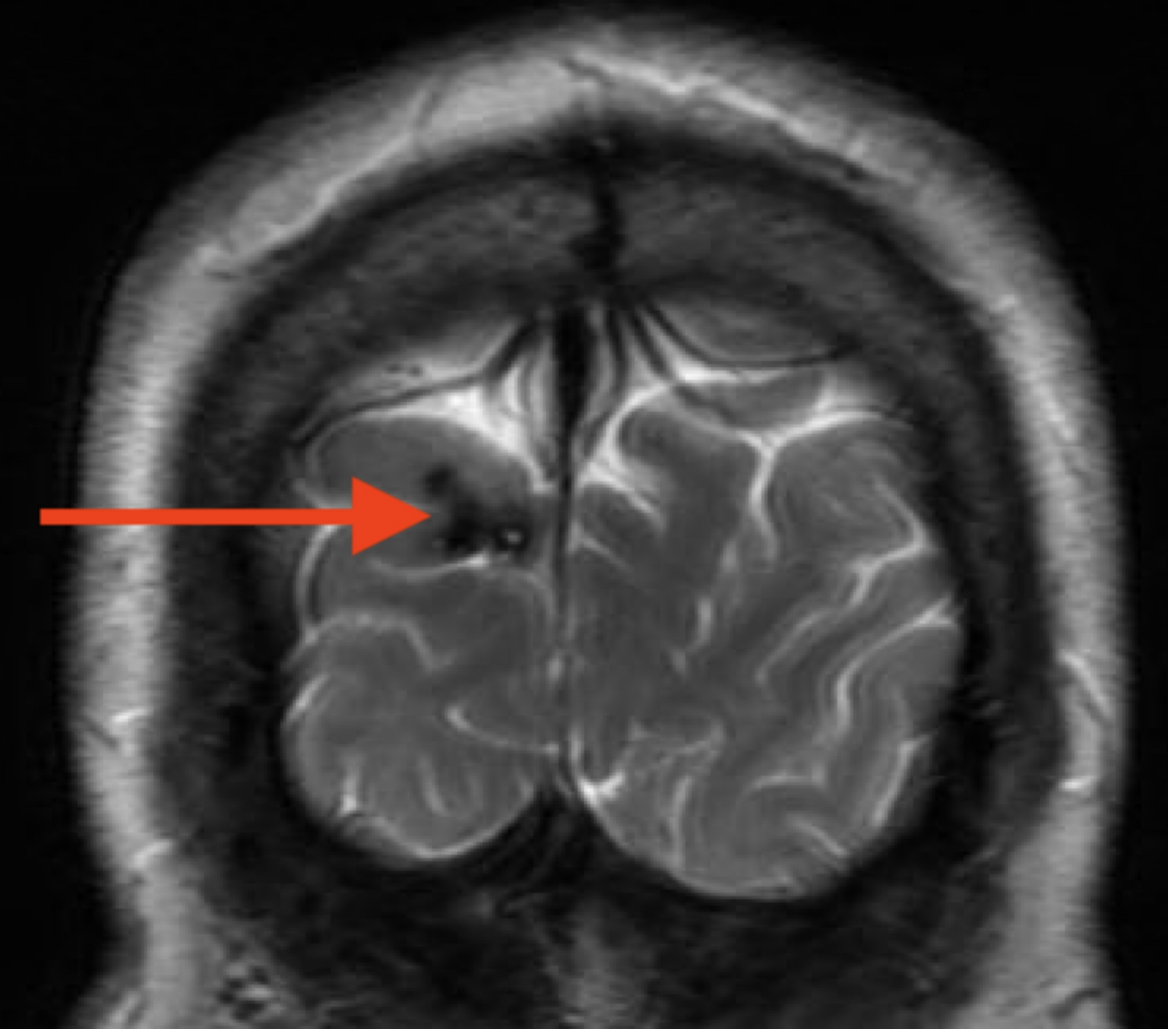
T2 MRI showing hemosiderin deposits in the right occipital lobe associated with cavernoma.

The patient had a past medical history of Factor V Leiden Mutation, recurrent PEs, and DVTs. Two months prior to presentation he complained of right frontal and perioral numbness and was subsequently diagnosed with a 1.3-cm bleed from multiple cavernous angiomas, located in the left midbrain and right occipital region (Figures 3-8). Consequently, his warfarin was discontinued, and an inferior vena cave (IVC) filter was placed while he was transferred to another facility for gamma knife radiation which was unsuccessful.

Considering the recent ICH and now multiple and recurrent PE in the setting of hereditary thrombophilia, there was an urgency for resuming anticoagulation. After a discussion with his neurosurgeon and a thromboembolism specialist, it was decided to restart anticoagulation. Prior to initiating anticoagulation, a CT scan was done to rule out any new lesion. He was started on a heparin drip and this was later transitioned to enoxaparin in case there would be a need for a reversal agent. The patient was discharged from the hospital in a stable condition and was transitioned to apixaban after a week. As of his follow-up appointments, first at one and then at four months after discharge, he was compliant with apixian and doing well, with no residual neurological deficits. The patient also had a follow-up CT scan of the head a year after discharge which showed no acute findings.

## Discussion

Factor V Leiden occurs due to a single point mutation of G-to-A transition at nucleotide 1691 in the factor V gene that leads to the amino acid substitution of arginine to glutamine [6]. As a result of the single amino acid substitution, procoagulant Factor Va becomes resistant to Activated Protein C (APC). APC is an anticoagulant protein that cleaves and inactivates procoagulant Factors Va and VIIIa. Due to this resistance factor Va is inactivated at a significantly slower rate than normal, resulting in increased thrombin generation and this is recognized as a cause of VTE [1,7].

Treatment for patients with Factor V Leiden depends on the clinical presentation. The first incidence of VTE should be treated with low molecular weight heparin (LMWH) or IV unfractionated heparin. Simultaneously, bridging should commence with warfarin to achieve an international normalized ratio (INR) of 2.5 as this therapy is sufficient even if the patient is homozygous for Factor V Leiden [8,9]. The duration of anticoagulation hinges on the risk factors for recurrent VTE and risk of anticoagulant induced bleeding. Approximately 30% of patients who suffer from VTE develop a recurrent event in the next 10 years [1]. A minimum of three months of anticoagulation is advised for patients with provoked VTE, while a longer course of anticoagulation is required for patients with a first or recurrent unprovoked VTE. While patients with homozygous Factor V Leiden mutation are at even greater risk of VTE these patients are occasionally started on long-term anticoagulation prophylaxis if only other risk factors are present [9,10].

Our patient was on long-term anticoagulation therapy due to his history of multiple recurrent VTEs. Once he suffered from ICH due to a cavernous angioma, it was judicious to hold anticoagulation as ICH in the presence of anticoagulation leads to death or disability in 76% of cases [11]. In the first 24 hours after ICH, the risk of hematoma expansion is very high, and it is considered not safe to administer anticoagulation. Immediate reversal of anticoagulation after ICH diagnosis is recommended. The risk of expansion of a hematoma is highest at presentation and is inversely related to time, however, the risk of thromboembolic events (especially VTE) is continuous and cumulative [12].

Unfortunately, the risk of ICH recurrence in patients receiving anticoagulation is not clear. Fong et al. showed in a recent study that increased ICH mortality was associated with patients on warfarin for atrial fibrillation, however, there are no population-based studies as of yet [13]. ICH recurrence risk in general is about 2% to 4% per patient-year, while the risk of suffering a major bleed on warfarin therapy is about 2% to 3% per year and may be higher in the first month. When deciding to restart anticoagulation we suggest that ICH should be differentiated between lobar and deep as they both carry different risks for recurrent bleeding [12]. Eckman et al. noted that use of oral anticoagulation in patients with a deep ICH resulted in 31 fewer VTE events per 1000 patients at the cost of 19 additional ICH. Comparatively, patients with a lobar ICH receiving oral anticoagulation resulted in 31 fewer VTE events per 1000 patients but at the cost of 150 additional ICH events in the first year of treatment [14].

Cerebral cavernous malformations (CCMs) represent a possible cause for intracranial hemorrhage as was the case in our patient. CCMs are characteristically defined as low pressure berrylike vascular lesions that generally lack neuronal innervation and smooth muscle cells. These thin-walled vascular malformations behave more like sinusoids and are big risk factors for hemorrhage [15]. It has been established that patients with a recent hemorrhage from a CCM are at increased risk for hemorrhage recurrence [16]. As a result, clinicians are cautious when considering antithrombotic therapy in CCM-related hemorrhage.

We report a case of a patient with a history of factor V-related VTEs, who developed a CCM-related ICH while receiving anticoagulation. It posed a therapeutic dilemma for our patient who required long-term anticoagulation therapy for recurrent VTEs due to underlying Factor V Leiden mutation. It was decided that despite the history of multiple PEs, anticoagulation therapy should be stopped acutely and an IVC filter was placed while treatment was pursued for the CCM. Current guidelines according to the American Heart Association suggest that anticoagulation could be resumed four weeks post-ICH but there seems to be a lack of consensus regarding this topic [17,18].

Due to this lack of consensus of when to resume anticoagulation we felt unsure of when to restart the patient’s anticoagulation after the CCM-related hemorrhage. The IVC filter proved to be insufficient as our patient was later diagnosed with multiple right-sided PEs. On repeat imaging of the brain and head, it was revealed that the patient had multiple cavernous angiomas in the occipital and temporal lobe.

Inferior vena cava filters are important in preventing PEs and are typically placed in patients with an acute DVT or PE when there is a contraindication to anticoagulation therapy. The use of IVC filters has significantly decreased over the last 10 years and surprisingly the PE rates have remained stagnant and even decreased per some data [19]. In a seven-year retrospective study by Somarouthu et al., results showed that IVC filter placement in stroke patients resulted in imaging confirmed PE at a frequency of 4% and post-filter imaging confirmed DVT occurred in 14% of patients. They concluded that IVC filters have an adequate safety profile and are efficient in preventing fatal PE in patients with stroke [20].

After gamma knife radiation surgery of the angiomas was unsuccessful, we made the decision to resume anticoagulation to prevent more PEs. The fact that the CCM-related ICH was from a deep source is a characteristic that made restarting anticoagulation a safe option. This case also highlights the need for systematic studies to better outline when to restart anticoagulation per specific risk factors (especially location) post ICH. While the American Heart Association suggests four weeks post ICH to resume anticoagulation, a meta-analysis by Zhou et al. suggested there is still no firm time table when to resume therapy and further studies are needed [18].

## Conclusions

Anticoagulation after a history of a hemorrhagic stroke poses a therapeutic dilemma. The unknown risk of rebleeding in a patient with an ICH versus the risk of another VTE event has to be weighed carefully and studied prospectively. Based on our case, ICH from a deep source is likely a better characteristic that favors a resumption of anticoagulation. This case highlights that IVC filters cannot solely be relied upon in a patient at high risk for thrombotic events. There is a need for a systematic study to analyze the risk of rebleeding in patients with a prior CCM-related hemorrhage, time table of when to resume anticoagulation following ICH, and risk stratification according to ICH location.
